# Hospital burden of critical illness across global settings: a point prevalence and cohort study in Malawi, Sri Lanka and Sweden

**DOI:** 10.1136/bmjgh-2024-017119

**Published:** 2025-03-25

**Authors:** Carl Otto Schell, Raphael Kazidule Kayambankadzanja,, Abi Beane, Andreas Wellhagen, Chamira Kodippily, Anna Hvarfner, Grace Banda, Nalayini Jegathesan, Christoffer Hintze, Wageesha Wijesiriwardana, Martin Gerdin Wärnberg, Jayasingha Arachchilage Sujeewa, Mtisunge Kachingwe, Petronella Bjurling-Sjöberg, Isaac Mbingwani, Annie Kalibwe Mkandawire, Hampus Sjöstedt, Wezzie Kumwenda-Mwafulirwa, Surenthirakumaran Rajendra, Odala Kamandani Dzinjalamala, Cecilia Stalsby Lundborg, Kwazizira Samson Mndolo, Miklós Lipcsey, Rashan Haniffa, Lisa Kurland, Markus Castegren, Tim Baker

**Affiliations:** 1 Global Public Health, Karolinska Institutet, Stockholm, Sweden; 2 Centre for Clinical Research Sörmland, Uppsala University, Eskilstuna, Sweden; 3 Department of Medicine, Nyköpings Hospital, Region Sörmland, Nyköping, Sweden; 4 Market Dynamics, Global Health Program, PATH, Lilongwe, Malawi; 5 Network for Improving Critical Care Systems and Training, Colombo, Sri Lanka; 6 Pandemic Science Hub and Institute for Regeneration and Repair, Edinburgh, UK; 7 Network for Improving Critical Systems and Training, Colombo, Sri Lanka; 8 Research, Education, Development & Innovation (REDI), Region Vastra Gotaland, Gothenburg, Sweden; 9 Adult Emergency and Trauma Centre, Queen Elizabeth Central Hospital, Blantyre, Malawi; 10 Emergency Medicine Unit, Kamuzu University of Health Sciences, Blantyre, Malawi; 11 Teaching Hospital Jaffna, Jaffna, Sri Lanka; 12 Department of Otorhinolaryngology, Karolinska University Hospital, Stockholm, Sweden; 13 Department of Allied Health Sciences, University of Colombo Faculty of Medicine, Colombo, Sri Lanka; 14 National Intensive Care Surveillance-MORU, Colombo, Sri Lanka; 15 Perioperative Medicine and Intensive Care, Karolinska University Hospital, Stockholm, Sweden; 16 DGH Monaragala, Monaragala, Sri Lanka; 17 Anaesthesia and Intensive Care, Queen Elizabeth Central Hospital, Blantyre, Malawi; 18 Public Health and Caring Sciences, Uppsala University, Uppsala, Sweden; 19 Dedza District Health Office, Dedza, Malawi; 20 Medical Surgical Nursing, Malawi College of Health Sciences, Blantyre, Malawi; 21 Food Safety and Health Research Centre, School of Public Health, Southern Medical University, Guangzhou, Guangdong, China; 22 Department of Anesthesia and Intensive care, Queen Elizabeth Central Hospital, Blantyre, Malawi; 23 Nursing Department, Adult Health, Kamuzu University of Health Sciences, Blantyre, Malawi; 24 Department of Community and Family Medicine, University of Jaffna Faculty of Medicine, Jaffna, Sri Lanka; 25 Department of nursing, Malawi college of Health Sciences, Blantyre, Malawi; 26 Ministry of Health, Lilongwe, Malawi; 27 Anesthesiology and Intensive Care, Department of Surgical Sciences, Uppsala University, Uppsala, Sweden; 28 Hedenstierna Laboratory, Department of Surgical Sciences, Uppsala University, Uppsala, Sweden; 29 Pandemic Science Hub and Institute for Regeneration and Repair, University of Edinburgh, Edinburgh, UK; 30 School of Medical Sciences, Örebro University, Orebro, Sweden; 31 CLINTEC, Karolinska Institutet, Stockholm, Sweden; 32 Muhimbili University of Health and Allied Sciences, Dar es Salaam, United Republic of Tanzania

**Keywords:** Epidemiology, Health policies and all other topics, Health services research, Infections, diseases, disorders, injuries

## Abstract

**Introduction:**

The burden of critical illness may have been underestimated. Previous analyses have used data from intensive care units (ICUs) only, and there is a lack of evidence about where in hospitals critically ill patients receive care. This study aims to determine the burden of critical illness among adult inpatients across hospitals in different global settings.

**Methods:**

We performed a prospective, observational, hospital-based, point prevalence and cohort study in countries of different socioeconomic levels: Malawi, Sri Lanka and Sweden. On specific days, all adult in-patients in the eight study hospitals were examined by the study team for the presence of critical illness and followed up for hospital mortality. Patients with at least one severely deranged vital sign were classified as critically ill. The primary outcomes were the presence of critical illness and 30-day hospital mortality. In addition, we determined where the critically ill patients were being cared for and the association between critical illness and 30-day hospital mortality.

**Results:**

Among 3652 hospitalised patients, we found a point prevalence of critical illness of 12.0% (95% CI 11.0 to 13.1), with a hospital mortality of 18.7% (95% CI 15.3 to 22.6). The crude OR of death of critically ill patients compared with non-critically ill patients was 7.5 (95% CI 5.4 to 10.2). Of the critically ill patients, 96.1% (95% CI 93.9 to 97.6) were cared for in the general wards outside ICUs.

**Conclusions:**

The study has revealed a substantial burden of critical illness in hospitals from different global settings. One in eight hospital in-patients was critically ill, 19% of the critically ill died in hospital, and 96% of the critically ill patients were cared for outside of ICUs. Implementing the most feasible and low-cost critical care in general wards throughout hospitals would impact a large number of high-risk patients and has the potential to improve outcomes across all acute care specialties.

**WHAT IS ALREADY KNOWN ON THIS TOPIC**

- Critical illness has been defined as ill health with vital organ failure and a high risk of imminent death if care is not provided.
- Provision of timely care to stabilise vital organ function of critically ill patients (critical care) can prevent mortality.
- The global burden of critical illness has not been accurately determined. Existing estimates have been based on data only from intensive care units (ICU), a high-cost resource with widely varying availability between settings, and have not included critically ill patients in other hospital wards and units.
- The global incidence of critical illness has been estimated to be 30–45 million adults, and the hospital mortality of critically ill patients in ICUs has been estimated to be 22%.

**WHAT THIS STUDY ADDS**

- In this purposive sample of eight hospitals at different socioeconomic levels, critical illness was common among adult inpatients (12% point prevalence).
- Critical illness had a high hospital mortality (19%).
- Critical illness was commonly found outside ICUs. A strikingly large majority of the critically ill (96%) were cared for in general wards.
- These findings indicate that there is a huge burden of critical illness, notably outside ICUs.

**HOW THIS STUDY MIGHT AFFECT RESEARCH, PRACTICE OR POLICY**

- Critical illness is a more important issue than previously thought.
- Researchers, clinicians, patient organisations and policymakers should enhance efforts to reduce mortality and morbidity in the large group of critically ill patients in hospitals.
- Such efforts should be focused across hospital wards and units, not just in ICUs.
- Implementing feasible low-cost critical care interventions throughout hospitals would impact a large number of high-risk patients and has potential to improve outcomes across all medical specialties.

## Introduction

 Critical illness is as a ‘state of ill health with vital organ dysfunction, a high risk of imminent death if care is not provided and the potential for reversibility’.^1^ Regardless of underlying diagnosis, critically ill patients require similar supportive actions to stabilise vital organ functions and prevent death. Such critical care interventions^1^ are needed wherever a critically ill patient is located.^2^ Although many effective critical care interventions are low cost and feasible,^3 4^ there is alarming evidence from different settings that they are frequently not provided.^5^,^8^ This may be an important contributor to the 8.6 million annual deaths estimated to be due to poor quality health services.^9^ Improving care for critically ill patients could increase survival across all acute care specialties.^10^,^15^

The World Health Assembly 2023 decided that critical care should be part of universal health coverage.^16^ Yet, there is limited evidence to inform policymakers about the burden of critical illness. Priority decisions and investments in healthcare and research are often grounded in sources based on patients’ diagnoses where information about critical illness is not captured.^17 18^ Most research into critical illness has been confined to intensive care units (ICUs), where advanced and high-cost critical care is provided.^19^ ICUs are sparse in rural and low resource settings where the needs for critical care are assumed to be highest.^1420^,^23^ Per 100 000 population, ICU beds vary—from 0.1 in Malawi (low-income country) and 2.3 in Sri Lanka (middle-income country) to 5.8 in Sweden and 35 in the USA (high-income countries).^24^,^26^

It has been estimated that the global incidence of critical illness among adults is 30–45 million per year, based on extrapolation from a North American ICU registry.^27^ This may be an underestimation as the adult incidence of sepsis alone has been assessed to 24 million per year.^28^ Additionally, there are indications that critically ill patients may often be cared for outside ICUs.^29^,^35^ There is a need of data from critically ill patients throughout hospitals, across ward types, specialties and socioeconomic levels. In this multicentre global study, the objective was to assess the burden of critical illness among hospitalised adults.

## Methods

### Study design and settings

This was a prospective, observational, hospital-based, point prevalence and cohort study in Malawi, Sri Lanka and Sweden. The study countries were chosen to include a low-income, a middle-income and a high-income country. Annual health expenditure (US$) per capita ranges from 33 in Malawi to 151 in Sri Lanka and 6915 in Sweden.^36^ Data collections took place in individual days between 2017 and 2019 in the eight public hospitals including primary and referral hospitals in each country (table 1). All participants had their vital signs examined for the presence of critical illness, and they were followed up for hospital mortality, censored at 30 days. We used the term *burden* of critical illness when referring to the impact of critical illness—both occurrence (prevalence) and consequence (mortality).

**Table 1:** Study countries and hospitals (data from hospital administrations and World Bank)

Country	Malawi	Sri Lanka	Sweden
Population*	20 405 000	22 181 000	10 486 000
GDP/capita* (US$)	645	3354	56 424
Life expectancy (at birth)*	63	76	83
Maternal mortality* (per 100 000 live births)	451	43	3
Annual health expenditure per capita* (US$)	33	151	6915
Hospital beds per 100 000 population*	130	420	210
ICU beds per 100 000 population*	0.1	2.1	5.7

Hospital	Queen Elizabeth Central Hospital	Chiradzulu District Hospital	Monaragala District General Hospital	Point Pedro Base Hospital	Örebro University Hospital	Mälar Hospital, Eskilstuna	Nyköpings Hospital	Kullbergska Hospital,Katrineholm
Hospital type	Government-run university hospital	Government-run primary hospital	Government-run secondary hospital	Government-run primary hospital	Government-run university hospital	Government-run secondary hospital	Government-run primary hospital	Government-run primary hospital
Immediatecatchment population† (n)	1 000 000	240 000	556 000	150 000	200 000	146 000	96 000	59 000
Total adult hospital beds† (n)	1150	240	460	221	402	254	122	57
ICU beds† (n)	8	0	6	0	14	8	6	0
Admissions per year† (n)	40 000	10 000	48 000	24 448	29 000	18 821	10 823	4250
Mean length of stay adult† (days)	‡	2†	2†	2†	4,4†	4.6†	4.2†	4.6†
Hospital mortality per admission†	7%†	15%†	‡	0.6%†	3.8%†	3.6%†	3.1%†	3.9%†

Characteristics of study countries and hospitals. The numbers in the table are not based on the study cohort.

* World Bank Open Data 2024.

† Administrative data from study hospital.

‡ Data unavailable.

GDP, gross domestic product; ICU, intensive care unit.

The principles of Good Clinical Practice were followed. The strengthening of the reporting of observational studies in epidemiology initiative was used in the design and reporting of the study (online supplemental file 1).

### Participants

In each hospital on the days of point prevalence assessment, all patients admitted for in-patient care in the wards above 18 years of age were included in the study. All participants who were able to, provided informed consent. For the validity of the study, we included patients with reduced consciousness in the absence of objection from the patient (verbal or non-verbal) or from the next of kin. We could not include patients who were in operating theatres or those who were absent and could not be found later in the day. The study excluded patients who were not admitted to hospital (neither had stayed, nor planned to stay overnight), women in active labour, patients with a primarily psychiatric reason for admission and moribund patients identified as *dying* by the attending nurse.

### Variables

Critical illness was defined as *a state of ill health with vital organ dysfunction, a high risk of imminent death if care is not provided and a potential for reversibility*^1^ and operationalised in the same way in all study settings by classifying a critically ill patient as someone with one or more severely deranged vital sign at the point prevalence examination. Such criteria are independent of ward type, specialty and diagnosis and are pragmatic for use in clinical practice. The parameters and *a priori* decided cut-offs for severely deranged vital signs are based on ‘danger signs’ that are triggers for clinical intervention used at Karolinska University Hospital (Sweden) and in Muhimbili National Hospital (Tanzania) (table 2).^32 35 37 38^

**Table 2:** Parameters and cutoffs for severely deranged vital signs—danger signs

		Danger signs
A	Airway sounds	Stridor or gurgling or snoring
B	Respiratory rate (per minute)Oxygen saturation (%)	<8 or >30<90
C	Heart rate (per minute)Systolic blood pressure (mm Hg)	<40 or >130<90
D	Conscious level	Glasgow Coma Scale<9

The primary study outcomes were the point prevalence of critical illness and the 30-day hospital mortality of patients with critical illness. In addition, we identified where in hospitals critically ill patients were cared for and we assessed the association between critical illness and 30-day hospital mortality.

### Data sources

Each hospital was assessed at least two times to control for seasonal variation. All hospital wards and units were visited, regardless of admitting specialty, to avoid selection bias. Teams of nurses and students of healthcare professions went from ward to ward to include all the hospital in-patients and assess their vital signs. A senior health professional or researcher supervised in each ward to ensure quality data collection. Prior to this, all data collectors had training and practice on research methods, ethics, study methods, equipment usage and standardised vital signs assessments. The equipment was quality tested before each data collection and included automatic blood pressure monitors, pulse oximeters and clocks. Abnormal vital signs were rechecked, and alternative methods were used if a vital sign could not be assessed (eg, using manual blood pressure measurement). The nurse-in-charge of the ward was notified immediately when a patient was identified as critically ill. The research team offered to document all vital signs for use in patient care.

We used field validated case report forms (CRF) in all settings (online supplemental file 2). Paper-CRFs and double data entry into the database were used in Malawi and Sweden. In Sri Lanka, we used electronic CRFs on handheld tablets that subsequently fed into the database. In Malawi and Sri Lanka, the clinical background information was extracted from the paper-based patient records and outcomes were collected through follow-up of records and hospital administrative data. In Sweden, these data were collected from the electronic medical records.

The patients’ hospital records were used for clinical information about age, sex, specialty and decision to not resuscitate (DNR) in case of cardiac arrest. ICU beds were classified per hospital definition. All other patients were classified as located in general wards. Some hospitals described some ward beds as providing a higher care intensity termed ‘high care beds’ or ‘high dependency unit (HDU)’ beds. However, the care and interventions available in such locations varied substantially between settings, which precluded a formal analysis.

### Study size

The study had the ambition to include as many patients as logistics would allow in hospitals at different levels in a low-income, a middle-income and a high-income countries. A pilot sample before the study start yielded a point prevalence of critical illness of 10% and a mortality of critical illness of 15%. We used these data to calculate that a minimum sample size of 1225 patients would be required to estimate the expected point prevalence with 2% units’ absolute precision and a 95% confidence level.

### Statistical methods

We used percentages to present point prevalence, hospital mortality and location of the critically ill. The association of critical illness and hospital mortality was assessed using ORs in crude logistic regression models and adjusted ORs (aOR) in models including prespecified potential confounders: age, sex and country. Missing data for a single vital sign were classified as not being a danger sign (the most common value), enabling use of the other vital signs to classify the patient’s critical illness status. Participants who were lost to follow-up or had missing data for age were excluded, to ensure that all analyses were based on the same cohort. A 95% CI was used in reporting findings. Stata SE V.16.1 (Stata Corp, College Station, Texas) was used for statistical analyses.

## Results

### Participants

A total of 3682 participants were included initially. A final cohort of 3652 participants was used for analyses, after exclusion of 20 patients who were lost to follow-up (18 from Malawi and two from Sri Lanka) and 10 patients from Malawi with missing data for age. Out of 21 912 expected data points for vital signs (6 per participant), 56 (0.3%) were missing.

### Descriptive data

Women comprised 2015 (55%) of all participants and 224 (51%) of the critically ill. The median age of the cohort was 58 years (IQR 34–75) and varied from 35 years (IQR 26–49) in Malawi to 73 years (IQR 61–82) Sweden. Among critically ill patients, the median age was and 61 years (IQR 37–76). The majority of patients were admitted to a medical ward: 1846 (51%) of all patients and 327 (74%) of the critically ill. Clinical characteristics of the cohort are presented in table 3 and online supplemental file 3.

**Table 3:** Participant characteristics

	All settings		Malawi		Sri Lanka		Sweden	
	All	Critical	All	Critical	All	Critical	All	Critical
Participants, n	3652	439	1107	204	723	43	1822	192
Death in hospital, n (%)	178 (4.9%)	82 (19%)	85 (7.1%)	42 (21%)	8 (1.1%)	6 (14%)	85 (4.7%)	34 (18%)
Female, n (%)	2015 (55%)	224 (51%)	653 (59%)	119 (58%)	436 (60%)	17 (40%)	926 (51%)	88 (46%)
Age, years (IQR)	58 (34–75)	61 (37–76)	35 (26–49)	38 (30–51)	41 (30–62)	56 (39–68)	73 (61–82)	76 (69–86)
Specialty, n (% per column)								
Medicine	1846 (51%)	327 (74%)	461 (42%)	143 (70%)	244 (34%)	24 (56%)	1141 (62%)	160 (83%)
OBG	653 (18%)	24 (5.5%)	283 (26%)	16 (7.8%)	260 (36%)	7 (16%)	110 (6.0%)	1 (0.5%)
Surgery	1151 (31%)	87 (20%)	362 (33%)	45 (22%)	218 (30%)	11 (26%)	571 (31%)	31 (16%)
Unknown	2 (<0.1%)	1 (<0.1%)	1 (<0.1%)	0	1 (<0.1%)	1 (2.3%)	0	0
DNR, n (% per column)	348 (10%)	81 (18%)	2 (0.2%)	2 (1%)	0	0	346 (19%)	79 (41%)
Level of care, n (% per column)								
ICU	52 (1.4%)	17 (3.9%)	5 (0.5%)	3 (1.5%)	7 (1.0%)	3 (7.0%)	40 (2.2%)	11 (5.7%)
General ward	3600 (99%)	422 (96%)	1102 (99%)	201 (99%)	716 (99%)	40 (93%)	1782 (98%)	181 (94%)

DNR, decision to not resuscitate in case of cardiac arrest; ICU, intensive care unit; IQR, interquartile range; OBG, obstetrics and gynaecology.

### Main results

Critical illness was present in 439 patients, corresponding to a point prevalence of critical illness of 12.0% (95% CI 11.0 to 13.1). The critically ill patients had a hospital mortality of 18.7% (95% CI 15.3–22.6). Of the critically ill patients, 96.1 (95% CI 93.9 to 97.6) were cared for in a general ward. Outcome data are presented in table 4.

**Table 4:** Critical illness: point prevalence, mortality and location of care

	All	Malawi	Sri Lanka	Sweden
Prevalence critical illness, % (95% CI)	12.0 (11.0–13.1)	18.4 (16.3–20.1)	5.9 (4.4–7.9)	10.5 (9.2–12.0)
Mortality critical illness, % (95% CI)	18.7 (15.3–22.6)	20.6 (15.6–26.7)	14.0 (6.3–28.3)	17.7 (12.9–23.8)
Location of critically ill patients, % (95% CI)				
ICU	3.9 (2.4–6.1)	1.5 (0.5–4.5)	7.0 (2.2–20.1)	5.7 (3.2–10.1)
General ward	96.1 (93.9–97.6)	98.5 (95.5–99.5)	93.0 (79.9–97.8)	94.3 (89.9–96.8)

CI, confidence interval; ICU, intensive care unit.

Among the 30 patients excluded from analysis due to missing data for mortality and age, eight (27%) patients were critically ill (8/28 (29%) patients in Malawi and 0/2 (0%) patients in Sri Lanka). Among the three critically ill patients with missing data for age in Malawi, one died (33%).

In the whole cohort, the association between critical illness and death was OR 7.5 (5.4–10.2). Two prespecified adjusted logistic regression models were used. In the model adjusted for age and sex, aOR was 7.3 (5.3–10.0). In the model adjusted for age, sex and country, aOR was 6.1 (4.4–8.4) (table 5, online supplemental file 3). The use of cubic splines to ensure that the association between age and death was not underestimated did not change the association between age and mortality in any model and so was not used.

**Table 5:** The association between critical illness and 30-day hospital mortality

	All	Malawi	Sri Lanka	Sweden
**Crude models**				
Critical illness, OR (95% CI)	**7.5(5.4–10.2)**p<0.001	**5.2(3.3–8.2)**p<0.001	**55.0 (10.7-282)**p<0.001	**6.7(4.2–10.6)**p<0.001
**Adjusted models**				
Critical illness, aOR (95% CI)	**7.3(5.3–10.0)**p<0.001	**5.3(3.4–8.5)**p<0.001	**41.1(7.8–217)**p<0.001	**5.5(3.4–8.8)**p<0.001
Age (1 year), aOR (95% CI)	1.01 (1.00–1.02)	1.01 (0.99–1.02)	1.02 (0.98–1.06)	1.04 (1.02–1.06)
Sex (male), aOR (95% CI)	2.0 (1.4–2.7)	2.7 (1.7–4.4)	2.5 (0.5–14)	1.6 (0.9–2.5)
Critical illness aOR (95% CI)	**6.1(4.4–8.4)**p<0.001			
Age, (1 year), aOR (95% CI)	1.02 (1.01–1.03)			
Sex, (male), aOR (95% CI)	2.0 (1.5–2.8)			
Country, aOR (95% CI)				
Malawi	6.8 (3.2–14.4)			
Sweden	2.4 (1.1–5.2)			
Sri Lanka	1 (reference)			

aOR, adjusted OR; CI, confidence interval; IQR, interquartile range; OR, odds ratio.

## Discussion

### Key results

In this prospective point prevalence and cohort study of all in-patients in eight hospitals from Malawi, Sri Lanka and Sweden, we found a substantial burden of critical illness. The point prevalence of critical illness was 12% and the critically ill patients had a hospital mortality of 19%. A large majority of the critically ill patients (96%) were cared for in general wards outside of the ICUs.

### Strengths and limitations

The prospective examination of all in-patients, regardless of diagnosis and location in the hospital, minimised the risk of selection bias and misclassification in the study. The quality of the data collection processes increased internal validity through high inclusion rates, data accuracy and few missing data points. Studying hospitals in a low-income, a middle-income and a high-income country enabled the inclusion of patients from settings with a large global variation. The feasible clinical criteria for identification of critical illness and the pragmatic data collection methods that were used enable replication in health facility audits and in large studies.

The study has limitations. First, the pragmatic criteria for critical illness may have missed high-risk patients whose vital signs were insufficiently deranged or who had been stabilised by healthcare interventions. Conversely, some patients with adapted physiology due to chronic disease may have been misclassified as critically ill. Second, we could not include patients in operating theatres, some of whom may have been critically ill. Third, we did not collect data in emergency units. For feasibility and less heterogeneity concerning the clinical situation, the study included in-patients only. Fourth, data were collected during working hours and the burden of critical illness may be different in weekends and at night.^39^ Fifth, the ethical imperative to inform the ward nurses about the patients who were critically ill might have led to improved care and reduced critical illness mortality of the study cohort. Last, the restricted number of countries and hospitals included in the study limits generalisation, but we do not have reason to think that the burden of critical illness would be markedly different in other hospitals.

### Interpretation

The study findings from different global settings confirm previous signals that critical illness may be common across hospitals and outside ICU—and a marker of higher danger than diagnoses in the centre of attention. The point prevalence of critical illness in our study is consistent with data that could be extracted from single-centre studies with other aims. Among hospital patients in Finland and Sweden, 8% and 12%–14%, respectively, had a severely deranged vital sign.^30 34 35^ In medical and surgical wards in Uganda, 12% of patients had a ‘critical’ modified early warning score of more than 5.^33^ Our results support previous indications of a substantial burden of critical illness across hospitals in different global contexts.^27^

The mortality of critically ill patients found is high compared with other patient groups and diagnoses of public and policy interest. For example, patients admitted for care in Swedish ICUs had a hospital mortality of 14%.^40^ Among patients with COVID-19 during the severe first wave of the pandemic in USA in 2020, 20% died in hospital.^41^ Admitted patients with acute myocardial infarction with ST-elevation in Sub-Saharan Africa, South Asia and North Europe had 30-day mortalities of 2.4%–5.0%, 1.0% and 0.4%, respectively.^42^,^44^ While each critically ill patient also has an underlying diagnosis in need of treatment, the findings from this study confirm that critical illness, identified in a pragmatic way using single severely deranged vital signs, is a high-risk condition (crude OR 7.5) across global settings.

Contrary to common belief, most critically ill patients were cared for in general wards outside ICUs. As countries have such large differences in the number of ICU beds—350 times more beds per 100 000 population in the USA than in Malawi^26^—the presence of critically ill patients in general wards might be assumed to be specific to low-income countries. This is neither supported by our results nor by previous research.^30 31 34^ Future strategies aiming at reducing mortality in acute care need to consider care processes for critically ill patients in the general wards.

There are likely explanations behind specific findings in each of the study countries. Sri Lanka, the middle-income country, had the lowest prevalence and mortality of critical illness. However, it had also the lowest mortality of non-critically ill patients (0.3%), which explains the high OR for critical illness in Sri Lanka. One reason behind this may be a higher number of hospital beds (420) per 100 000 population than Sweden (210) and Malawi (130).^36^ This could lead to a lower threshold for admitting patients to hospitals in Sri Lanka than the other countries, thus ‘diluting’ the proportion of critically ill patients. It was not surprising that the highest critical illness mortality would be found for the patients in Malawi (21%) where resources are far scarcer than in the other countries, affecting both the determinants of health and the budget for healthcare.^36^ Additionally, critical illness may be more common outside hospitals, since limited access to health facilities may delay or preclude care in low-resource settings.^45^ The high mortality of critically ill patients in Sweden (18%) was an interesting finding and is most likely explained by the high median age of the patients (73 years). Frailty, multimorbidity and death are more common in older individuals.^46^ It should be noted that most patients with a DNR in Sweden receive active treatments for their acute illness, such as pneumonia or hip fracture, so they were included in analyses. Conversely, moribund patients were excluded from the study.

### Implications

The findings indicate the importance of health systems recognising and prioritising critical illness, not only in ICUs and emergency units but also throughout in-hospital services, units and wards. This would be an achievable aim and in fact, critical care for most critically ill patients can be provided outside ICUs.^56 10 12 14 47^,^49^ The recently defined Essential Emergency and Critical Care (EECC) describes such care and includes 40 foundational interventions selected for clinical effectiveness and feasibility in all hospital settings such as triage, airway protection, oxygen therapy and effective communication.^3 13^ EECC aligns with WHO’s Fair Choices for universal coverage, aiming to maximise equity, effectiveness and population impact of healthcare interventions.^50^ Ward-based critical care has lower costs and has been shown to be more cost-effective than care in ICUs for many patient groups.^51^,^54^ In Tanzania, EECC has been estimated to be highly cost-effective at 14 US$ per healthy life-year gained.^23^ Ensuring EECC is provided to all patients who need it throughout hospitals and health systems could be the rational first step when improving critical care services.^12 49^ In cases where such foundational critical care is not enough to stabilise organ functions, HDUs may be a reasonable subsequent step for the health system and may be more equitable and effective than focusing on expansion of resource-intensive ICUs.^1555^,^57^ In current times, when critical care is becoming part of universal health coverage,^16^ governments and health organisations that use a strategy to improve critical care of starting from the foundational level and building up from there could reach all critically ill patients, be cost-effective, and maximise impact at population level.^11 12 15^

## Conclusion

The study has revealed a substantial burden of critical illness in hospitals from different global settings. One in eight hospital in-patients was critically ill, 19% of the critically ill died in hospital, and 96% of the critically ill patients were cared for in general wards outside of ICUs. Implementing feasible, low-cost, critical care in general wards and units throughout hospitals would impact a large number of high-risk patients and has the potential to improve outcomes across all acute care specialties.

## Supplementary material

10.1136/bmjgh-2024-017119 online supplemental file 1

10.1136/bmjgh-2024-017119 online supplemental file 2

10.1136/bmjgh-2024-017119 online supplemental file 3

## Data Availability

Data are available upon reasonable request.
