# Supplementary material for: Hospital burden of critical illness across global settings: a point prevalence and cohort study in Malawi, Sri Lanka and Sweden
Source: BMJ Glob Health. 2025 Mar 25;10(3):e017119. doi: 10.1136/bmjgh-2024-017119 (PMC12004492; doi:10.1136/bmjgh-2024-017119)
Supplement: online supplemental file 2 [file bmjgh-10-3-s002.pdf]

CRF

Hospital

CRISPOS
Critical Illness and Sepsis Prevalence and Outcomes Study

**Study number** Team/patient eg A1/001	__ __/__ __ __	**Ward**		**HDU?**	Y ☐ N ☐	**Date**	dd / mm / yyyy __/__/____	**Time**	hh . mm __ __.__ __ am ☐ pm ☐

FROM NOTES

**Patient Name**		**Hospital number**		**Age** (years)		**Sex**	**M☐ F☐**

**Date admitted to hospital**	dd / mm / yyyy __/__/____	**Time admitted to hospital**	hh . mm __ __.__ __ am☐ pm☐
**Acute Diagnosis Primary** (main reason for admission)		**Specialty**	
**Other Acute Diagnoses** (other or differential)	1. 3.	2. 4.	
**Chronic Diagnoses**	1. 3.	2. 4.	
**Had surgery in hospital?** (this admission)	**Y☐ N☐**	**If yes, what operation?**	

**Receiving Antibiotic?*** (prescribed in notes)	**Y☐ N☐**	**Receiving Antimalarial?**** (prescribed in notes)	**Y☐ N☐**	**Serum Lactate** (result from last 6 hrs in notes)	…………………..mmol/l unknown ☐

* **Antibiotics:** amoxycillin, ampiciilin, ceftriaxone, chloramphenicol, ciprofloxacin, co-trimoxaole, gentamycin, meropenem, metronidazole, x-pen or any other antibiotic
** **Antimalarials:** artesunate, LA, quinine or any other antimalarial

FROM PATIENT

**Position of patient**	**Lying flat on back** (<30°) ☐ **Lying on side**☐ **Head-up** (30-60°) ☐ **Sitting** (>60°) ☐ **Head-down** ☐ **Other** ☐ ……………………………………….
**Airway sounds** (listen standing by bedside)	**Clear** ☐ **Snoring**☐ **Gurgling**☐ **Stridor**☐

**GCS**	**Eyes**		**Verbal**		**Motor**		**Total**
	4 = opens spontaneously<br>3 = opens to voice<br>2 = opens to pain<br>1 = does not open eyes	/4	5 = normal<br>4 = disorientated<br>3 = words, incoherent<br>2 = incomprehensible sounds<br>1 = no sounds	/5	6 = normal<br>5 = localizes pain<br>4 = withdraws from pain<br>3 = flexes from pain<br>2 = extension to pain<br>1 = no movement	/6	/15
**AVPU** (**A**lert, responds to **V**oice, responds to **P**ain or **U**nresponsive)					**A** ☐ **V** ☐ **P** ☐ **U** ☐		

**Heart rate**	/minute	**Systolic BP**	/mmHg
**Temperature**	°C	**Diastolic BP**	/mmHg
**Cold hands?**	**Y☐ N☐**	**Capillary Refill Time**	<2 secs ☐ >2 secs☐
**Respiratory Rate**	/minute	**Oxygen Saturation**	%

**Receiving IV fluids** (bag attached to patient now)	**Y☐ N☐**	**If yes – state which**	Ringers Lactate ☐ Normal Saline ☐ 5% dextrose ☐ Other (write) ☐ ………………………		
**Receiving Oxygen** (now)	**Y☐ N☐**	**Receiving IV vasopressor***** (now)	**Y☐ N☐**	**Airway Action****** (now)	**Y☐ N☐**

*** **Vasopressors**: noradrenaline, adrenaline, dopamine or dobutamine ****Airway Action: Chin lift, jaw thrust, oro-pharyngeal airway, naso-pharyngeal airway, intubated

**Ability to walk** (ask patient to stand up and walk)	**Independently** ☐ **With assistance** ☐ **Unable** ☐ **Refuses** ☐

**Any notes or comments from data collection**	……………………………………………………………………………………………………… ………………………………………………………………………

CRF

**Outcome**	Died ☐<br>Discharged ☐		
**Date of death/discharge**	dd / mm / yyyy __/__/____	**Time of death/discharge**	hh . mm __.__ am☐ pm☐
