# Supplementary material for: Hospital burden of critical illness across global settings: a point prevalence and cohort study in Malawi, Sri Lanka and Sweden
Source: BMJ Glob Health. 2025 Mar 25;10(3):e017119. doi: 10.1136/bmjgh-2024-017119 (PMC12004492; doi:10.1136/bmjgh-2024-017119)
Supplement: online supplemental file 3 [file bmjgh-10-3-s003.pdf]

Supplementary file 3: Primary Diagnosis

Table S1. Diagnosis group based on International Classification of Diseases (ICD) per country and critical illness status.

Diagnosis group	All		Malawi		Sri Lanka		Sweden	
	All	Critical	All	Critical	All	Critical	All	Critical
Any diagnosis	3652	439	1107	204	723	43	1822	192
Circulatory	437	76	53	21	56	6	328	49
Dermatological	54	6	19	4	18	2	17	0
Ear, Nose and Throat	30	3	9	3	11	0	10	0
Endocrine, Nutritional, Metabolic	132	7	17	2	58	2	57	3
Gastrointestinal	380	38	79	21	88	6	213	11
Infectious	281	62	155	45	53	7	73	10
Musculo-skeletal	173	7	16	1	19	1	138	5
Neurological	193	23	56	12	32	3	105	8
Obstetric and Gynaecological	388	14	219	13	90	1	79	0
Oncologic and haematological	436	58	164	36	11	0	261	22
Ophthalmological	58	0	40	0	0	0	18	0
Respiratory	267	81	53	20	47	6	167	55
Trauma and Intoxication	455	36	153	19	87	3	215	14
Urogenital	241	16	48	2	104	4	89	10
Other	127	12	26	5	49	2	52	5

Logistic regression model adjusted for diagnosis groups

In the whole cohort, the crude association between critical illness and death was OR 7.5 (5.4-10.2). In the model adjusted for age, sex, country and diagnosis groups the aOR was 5.1 (3.5-7.2).
